# Neocortical development as an evolutionary platform for intragenomic conflict

**DOI:** 10.3389/fnana.2013.00002

**Published:** 2013-04-09

**Authors:** Eric Lewitus, Alex T. Kalinka

**Affiliations:** Max Planck Institute of Molecular Cell Biology and GeneticsDresden, Germany

**Keywords:** parent-offspring conflict, evolution, neocortex, genomic imprinting, X-chromosome, development, mammals

## Abstract

Embryonic development in mammals has evolved a platform for genomic conflict between mothers and embryos and, by extension, between maternal and paternal genomes. The evolutionary interests of the mother and embryo may be maximized through the promotion of sex-chromosome genes and imprinted alleles, resulting in the rapid evolution of postzygotic phenotypes preferential to either the maternal or paternal genome. In eutherian mammals, extraordinary *in utero* maternal investment in the brain, and neocortex especially, suggests that convergent evolution of an expanded mammalian neocortex along divergent lineages may be explained, in part, by parent-of-origin-linked gene expression arising from parent-offspring conflict. The influence of this conflict on neocortical development and evolution, however, has not been investigated at the genomic level. In this hypothesis and theory article, we provide preliminary evidence for positive selection in humans in the regions of two platforms of intragenomic conflict—chromosomes 15q11-q13 and X—and explore the potential relevance of *cis*-regulated imprinted domains to neocortical expansion in mammalian evolution. We present the hypothesis that maternal- and paternal-specific pressures on the developing neocortex compete intragenomically to influence neocortical expansion in mammalian evolution.

## Parent-offspring conflict at the fetal-placental interface

Development of the fetal-placental interface, which gives the fetus direct access to manipulate the availability of maternal resources, is a cardinal event in the evolution of viviparous mammals. Furthermore, while viviparity has evolved in other vertebrate taxa (e.g., Squamata), the transition from oviparity (egg-laying) to viviparity has been institutionalized in mammals, such that no species has transitioned back to oviparity in 200 million years (Killian et al., 2001a,b; Meredith et al., 2011). Importantly, evolution of the fetal-placental interface in mammals has established a unique genomic platform for conflict, or sometimes coadaptation (Curley et al., 2004; Wolf and Hager, 2006), between the fetal and maternal genomes. And the munitions employed in that conflict are genomic imprinting and X-linked gene expression.

Imprinted genes are inherited biparentally in the fetal genome, but during gametogenesis these genes are epigenetically modified to express only one parental allele in zygotic development (Li et al., 1999; Reik and Walter, 2001). In most cases, imprinted genes occur in a cluster (Williamson et al., 2006) and are controlled by a tissue- and temporal-specific *cis*-acting regulatory element, the so-called imprinting control element (ICE), that is imprinted on a parental-specific allele by DNA methylation or histone modification (Santoro and Barlow, 2011). Maintenance of the imprint is regulated in the embryo by either CTCF-dependent insulators or long non-coding RNAs (Sleutels and Barlow, 2002; Lewis and Reik, 2006; Mancini-Dinardo et al., 2006; reviewed in Bartolomei and Ferguson-Smith, 2011), the deletion of which results in regional loss of imprinting (Wutz et al., 1997; Thorvaldsen et al., 1998; Fitzpatrick et al., 2002; Lin et al., 2003; Williamson et al., 2006). While not all viviparous taxa show evidence for genomic imprinting (e.g., species of fish), imprinting is ubiquitous in viviparous mammals (Lawton et al., 2005; Renfree et al., 2009). The deep conservation of viviparity and imprinting in mammals emphasizes both the profound integration of the fetus into the apportioning of maternal resources through the maternal-fetal interface, as well as the considerable inclusive-fitness asymmetry in the contribution of parental resources. Importantly, neurological diseases associated with imprinted domains show divergent symptoms depending on the parent-of-origin of the imprinted allele (Crespi, 2008; Ubeda and Wilkins, 2008).

The phenomenon of conserved genomic imprinting in mammals has been subsumed into a more general theory of mammalian evolution, Parent-Offspring Conflict (Trivers, 1974; Moore and Haig, 1991; Wilkins and Haig, 2003). This theory describes, with formidable accuracy, how maternal and paternal interests, particularly in polygamous societies, may compete intragenomically over the allocation of resources to the fetus. That is, when a gene affects the fitness of an individual to whom the maternally inherited and paternally inherited alleles have different degrees of relatedness, the optimal strategy of each allele will depend on its parent-of-origin. In the case of multiple paternity, the paternal genome is more concerned with maximizing maternal resource allocation to the fetus at the expense of future (unrelated) progeny, whereas the primary interest of the maternal genome is to allocate resources evenly to all (equally related) progeny. Therefore, genes involved in parent-offspring conflict and the allocation of maternal resources should overlap with genes prone to evolving imprinted expression. Unsurprisingly, loci that influence embryonic growth (e.g., *Igf2, Stox1*, and genes involved in secreting placental lactogens) and cell division (e.g., *Aspm, Erbb3*) are often associated with imprinted genes (Haig, 1993; Tycko and Morison, 2002; Reik et al., 2003; Constância et al., 2004; Oudejans et al., 2004; Arngrímsson, 2005; van Dijk et al., 2005; Singhmar and Kumar, 2011). In the case of the brain, and the mammalian neocortex in particular, the involvement of imprinting centers of the latter type may be especially important for their effect on neurogenesis for two reasons: mammals with relatively high metabolic rates do not produce relatively large-brained progeny (McNab, 1989; Harvey and Krebs, 1990), as would be expected if brain size were determined strictly by maternal metabolic rates and access to maternal nutrient transfer (Lewitus et al., 2012b); and genomic loci governing variation in brain and body size are largely independent (Hager et al., 2012), so evolutionary changes in brain size cannot be primarily arbitrated by embryonic growth factors, but must be responsive to brain size-specific selection pressures decoupled from overall embryonic growth. Here, we discuss two genomic platforms for parent-offspring conflict in neocortical evolution: genomic imprinting at human chromosome 15q11-q13 and the human X-chromosome. We focus specifically on brain-expressed genes and, based on a review of the literature as well as preliminary analyses presented here, propose a major role for genomic imprinting and X-linked gene expression, regulated by *cis*-acting long non-coding RNAs, in the evolution of an expanded mammalian neocortex.

### Human chromosome 15q11-q13

A disproportionate number of imprinted genes are expressed, sometimes exclusively, in the brain (Wilkinson et al., 2007). While it was, at first, indicated that maternally inherited genes showed a bias for expression in the cortex and paternally inherited genes showed a bias in the hypothalamus (Keverne et al., 1996), recent evidence has demonstrated this not to be the case. In fact, 70% of imprinted genes in the preoptic area and medial prefrontal cortex of the mouse show a bias for expression of the paternal allele (Gregg, 2010). On human chromosome 15q11-q13, several brain-specific genes are expressed exclusively from the paternally inherited allele and only two genes, *Ube3a* and *Atp10a*, are expressed from the maternally inherited allele (Chamberlain and Lalande, 2010); these paternally and maternally inherited genes are controlled by the Prader-Willi Syndrome (PWS) and Angelman Syndrome (AS) imprinting centers, respectively (Saitoh et al., 1996). *Ube3a* is present in the nucleus and cytoplasm, observed only in neurons, and can ubiquitylate the DNA-repair and cell-cycle progression proteins HHR23 and MGMT, thereby targeting their destruction (Reis et al., 1994; Albrecht et al., 1997; Kumar et al., 1999; Srivenugopal and Ali-Osman, 2002; Le Meur et al., 2005; Dindot et al., 2008). It has recently been shown that *Ube3a* interacts with the microcephaly-related gene *Aspm*, possibly by regulating HHR23 in S-phase, and that knockdown of *Ube3a* leads to increased apoptosis due to chromosome missegregation and abnormal spindles (Singhmar and Kumar, 2011). In normal eutherian development, maternal expression of *Ube3a* is regulated by the paternally inherited long non-coding antisense RNA (LNCAT), *Ube3a-ATS*, that encompasses *Snurf-Snrpn, Snord116, Snord115*, and extends to *Ube3a* (Rougeulle et al., 1998; Runte et al., 2001; Landers et al., 2004; Rapkins et al., 2006). Because the AS imprinting center represses the PWS imprinting center on the maternal allele (Brannan and Bartolomei, 1999), when the former is mutated or deleted the latter loses its DNA methylation imprint. This results in the epigenetic silencing of the maternal *Ube3a* and causes neurodevelopment to proceed without ubiquitin ligase, ultimately leading to AS, a heritable disorder characterized by microcephaly (Kishino et al., 1997; Yamasaki et al., 2003). Likewise, when the paternal PWS imprinting center is mutated or deleted, neurodevelopment is disrupted, resulting in PWS, a disorder complemented by, among other pathologies, cognitive impairment (Cassidy and Driscoll, 2009).

### Human X-chromosome

The X-chromosome has evolved a preferential role in regulating brain development (Nguyen and Disteche, 2006; Swingland et al., 2012). This is evident, not only from genomic imprinting, but also in the evolution of brain-specific X-linked genes. The case of X-linked genomic imprinting is distinct from autosomal imprinting, because X-chromosome inheritance is biased by the sex of the fetus. That is, traits conferred by paternally expressed X-linked genes will only be heritable in females, whereas those conferred by maternally expressed X-linked genes will be heritable by both males and females. Nonetheless, effects of genomic imprinting in 45,X^m^ (single X-chromosome of maternal origin) and 45,X^p^ (single X-chromosome of paternal origin) individuals have proven to be considerable (Davies et al., 2006) and may, furthermore, reveal biases in the inheritance patterns of paternal versus maternal traits. 45,X^m^ individuals, for example, show larger superior temporal gyrus volume, but reduced gray matter in the caudate nuclei and white matter in the temporal lobes compared to 45,X^p^ individuals (Skuse et al., 1997). An explanation for this difference has implicated the *Xlr* gene family, which is imprinted with spatial- and temporal-specificity in the brain. Specifically, although evidence is scant, *Xlr* genes may influence brain plasticity during development by facilitating the rearrangement of protocadherins, genes that mediate cell adhesion, cytoskeletal organization, and morphogenesis, and are widely expressed throughout the brain (Frank and Kemler, 2002). Interestingly, only a subset of *Xlr* genes are imprinted in mouse compared to human, despite high sequence conservation (Davies et al., 2006).

Evidence for X-chromosome significance in regulating brain development is shown in its ability to disrupt neurodevelopment and the resulting pathologies. Fragile X syndrome (FXS) is a common form of inherited mental retardation (Laxova, 1994; Penagarikano et al., 2007). It is caused by the transcriptional silencing via hypermethylation of the 5′ UTR of Fragile X Mental Retardation 1 (FMR1), a RNA-binding protein involved in localized translation of target mRNAs (Fu et al., 1991; Oberle et al., 1991; Verkerk et al., 1991). The FMR1 protein is especially enriched in neurons and has been linked to the regulation of neural stem and progenitor cell proliferation during neurogenesis (Abitbol et al., 1993; Devys et al., 1993; Hinds et al., 1993; Callan and Zarnescu, 2011). FMR1-deficient mice show a 3-fold increase in neurons, including an increased population of Tbr2+ cells in the ventricular zone (VZ) and subventricular zone (SVZ) at embryonic day 17 (Castrén et al., 2005) and neural progenitor cells in cortical layers adjacent to the lateral ventricle (Tervonen et al., 2009), resulting in increased cell density in layer V of the postnatal neocortex. Likewise, FXS human embryos show a 5-fold increase in neurons, as well as notable gene expression changes, including an upregulation of *Erbb3* and the maternally imprinted *Diras3* (Bhattacharyya et al., 2008) and an enlargement of the caudate nuclei (Reiss et al., 1995; Eliez et al., 2001; Lee et al., 2007; Hoeft et al., 2008; Hazlett et al., 2009). Saffary and Xie (2011) recently showed that FMR1 utilizes an actin-dependent mechanism to control the ratio of radial glia (RG) and intermediate progenitor cells (IPs) during neurogenesis, suggesting that the observed increase in neurons is due to a shift in the relative ratio of RG to IPs during neocortical development. Evidence that FMR1 is involved in the translational control of *Tbr2* may also help explain why FMR1-deficiency leads to increased SVZ density in mice (Tervonen et al., 2009) and enlarged ventricle size in humans (Mostofsky et al., 1995; Reiss et al., 1995).

## Neurogenic gene expression and evidence for selection in imprinted domains

As a preliminary investigation into whether parent-of-origin-linked genes may play a role in neocortical evolution, we tested several selection models on imprinted genes, in addition to their long intergenic non-coding neighbors (lincRNAs), that are differentially expressed during human and/or mouse cortical neurogenesis.

### Pervasive purifying selection in imprinted and X-linked neurogenic genes

We retrieved RNA-seq data sampled prenatally from mouse and human neocortical germinal zones via the Gene Expression Omnibus (accession no. GSE38805) and analyzed differentially expressed coding (Anders and Huber, 2010) and non-coding (Cabili et al., 2011) transcripts. Imprinting status was determined for 49 differentially expressed genes at www.geneimprint.com (see Table A1). We collected multiple alignments of nucleotide sequences for at least 30 species, including one non-mammalian (*Gallus gallus*) and/or non-eutherian (*Macropus eugenii, Monodelphis domestica*, and/or *Ornithorhynchus anatinus*) outgroup, for the 49 imprinted genes, FXS-related *Erbb3*, and paternally imprinted *Usf1*, a transcription factor that interacts with the FMR1 promoter (Kumari and Usdin, 2001). Gene trees were constructed using a maximum-likelihood approach (Phylip v3.69) and analyzed in HyPhy (v2.1.2), which uses a branch-site model for detecting episodic diversifying selection along lineages (Kosakovsky Pond et al., 2011) and codon-based maximum-likelihood to estimate dN/dS at specific codon alignments (Kosakovsky Pond, 2005). These tests assume that substitutions can be described by a parameterized continuous-time Markov process and thereby model episodic selection in the substitution rates along lineages that may vary across both genomic sites (i.e., codons) and phylogenetic branches (Anisimova and Yang, 2007; Anisimova and Kosiol, 2009; Delport et al., 2010a,b; Yang and dos Reis, 2011).

**Figure 1:**
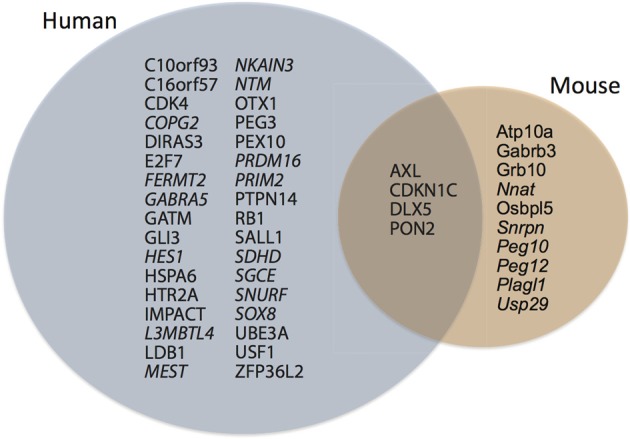
**During cortical neurogenesis, 38 genomically imprinted genes are differentially expressed in human germinal zones, whereas 14 are differentially expressed in mouse germinal zones.** Note that only four genes are expressed in both species, at least three of which are paternally imprinted. Italic type-face, maternally imprinted.

**Figure 2:**
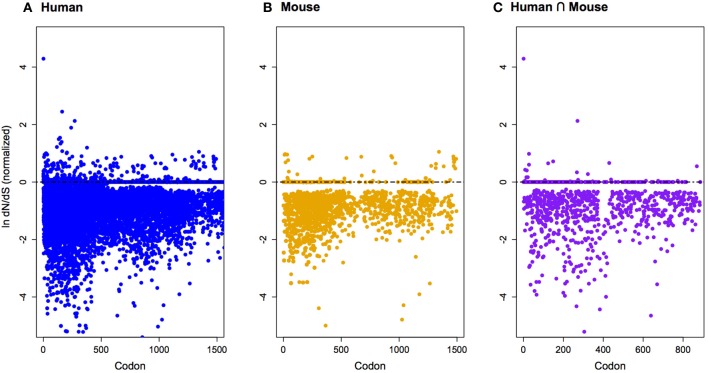
**Normalized ln-transformed dN/dS scores at each codon site for imprinted genes differentially expressed during cortical neurogenesis in only human (A), only mouse (B), and both human and mouse (C).** Note that genes are predominantly under pervasive purifying selection. See Table A1 for a list of genes.

**Figure 3:**
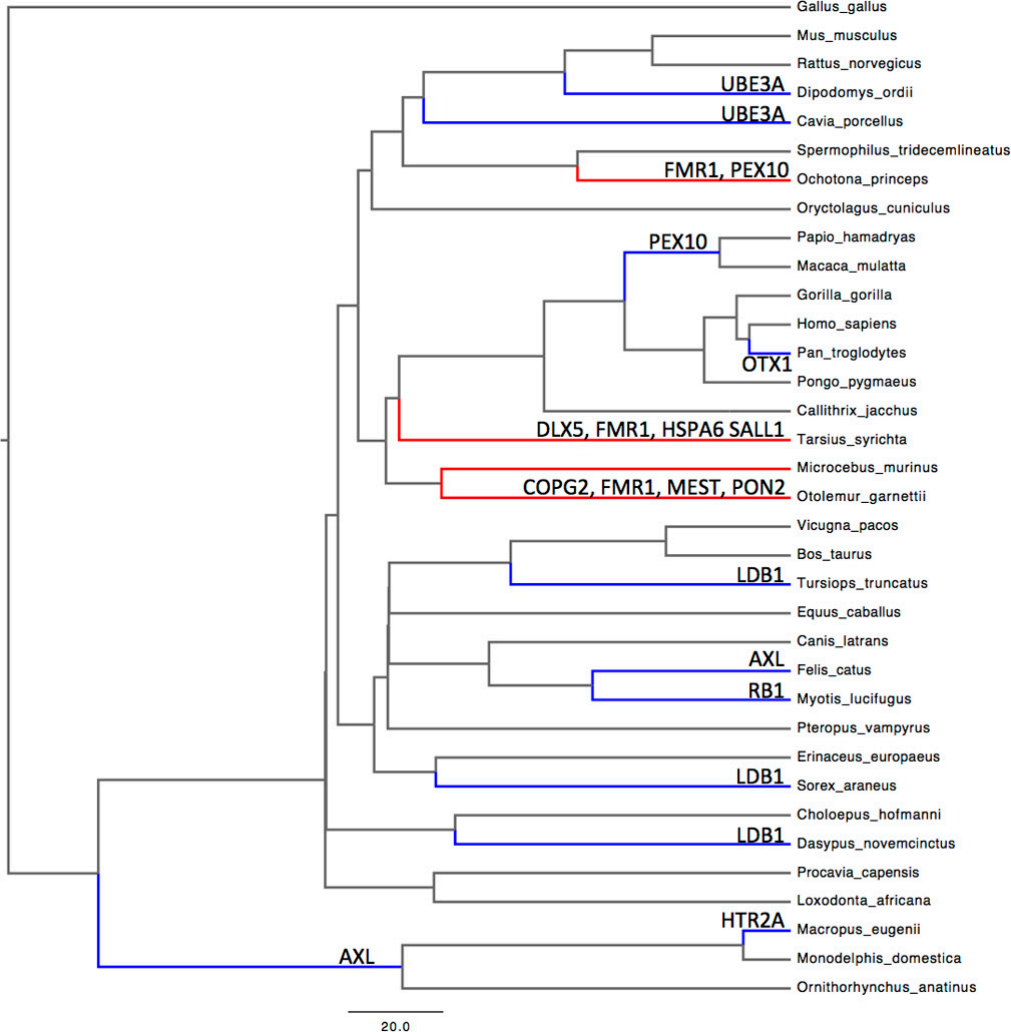
**Phylogeny of 34 mammalian and 1 non-mammalian vertebrate species.** Evidence of lineage-specific episodic selection in dN/dS substitution rates (*P* < 0.01) for imprinted neurogenic genes are listed on relevant branches. Red, evidence for selection in more than one gene along a single branch; blue, evidence for selection in a single gene. See Table A1 for a list of genes.

**Figure 4:**
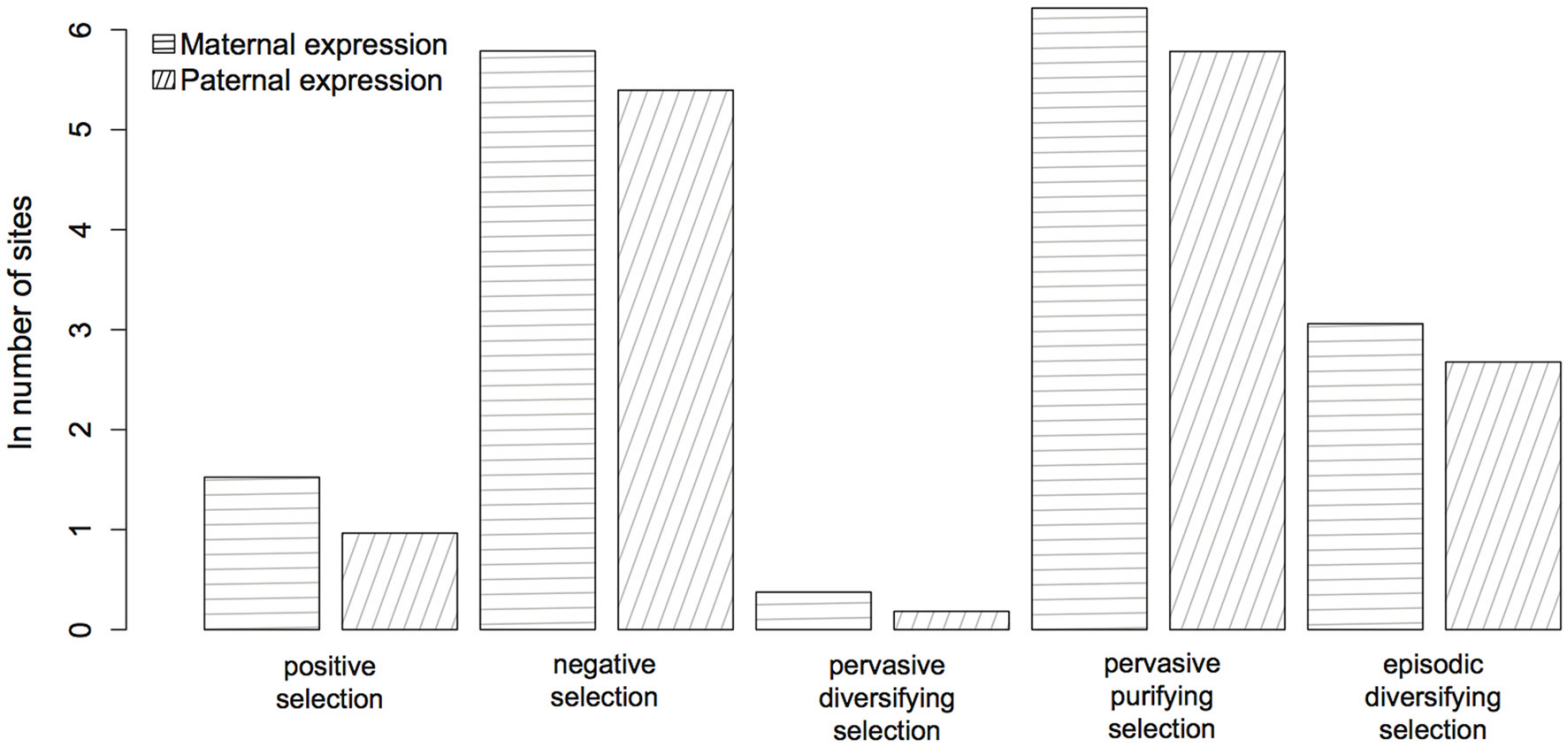
**Evidence for selection in 51 maternally (horizontal lines) and paternally (oblique lines) expressed genes (see Table A1).** The parent-of-origin of the expressed allele has no significant affect (*P* ≥ 0.07) on the number of selected sites.

The protein-coding genes investigated (Figure 1; Table A1) were predominantly under pervasive purifying selection in both mouse and human (Figure 2). In total, 29% of genes were found to have undergone diversifying selection along one or more branches (Figure 3). According to our selection criteria, no significant differences were found between maternally and paternally imprinted genes (Figure 4). The observed level of purifying selection in these genes is consistent with previous work showing disease genes, both complex and Mendelian, to be under greater purifying selection, based on substitution rate (dN/dS) as well as single nucleotide polymorphism data (Pn/Ps), than non-disease genes (Blekhman et al., 2008; Cai et al., 2009). That any disruptive mutation in these genes leads to severe disease phenotypes may explain both their intolerance to any deleterious mutations and strong need to be under pervasive purifying selection. This hypothesis, of course, does not take into account the considerable interspecific diversity in cortical organization (Butti et al., 2011; Lewitus et al., 2012a), but rather relates directly to neuron generation, a process that may be considered largely conserved in mammals (Franco and Müller, 2013). It moreover highlights the importance of non-coding regions, located in or nearby the imprinted domains, to differentially regulate the expression patterns of parent-of-origin-specific alleles in different species.

### Selective constraints in intergenic non-coding regions of imprinted domains

**Figure 5:**
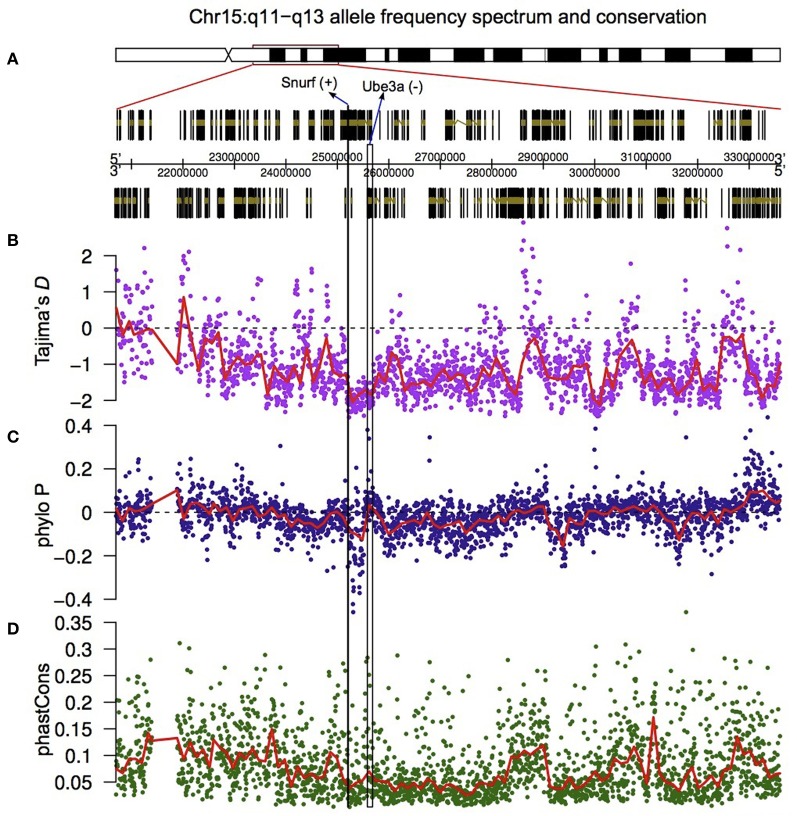
**Valley of selective constraint in the non-coding region located between *Ube3a* and *Snurf*.** The region on chromosome 15q11-q13 that has been explored at the sequence level **(A)**, Tajima's *D* measured in a 10 Kb sliding window across the highlighted region (with 2 Kb overlaps) **(B)**, and the PhyloP **(C)** and PhastCons **(D)** measures of nucleotide conservation.

On human chromosome 15q11-q13 the LNCAT *Ube3a-ATS* encompasses *Snurf* and *Ube3a* and represses expression of the paternally inherited copy of *Ube3a*. Three lincRNAs reside in this region, one of which exhibits upregulated expression in human embryos relative to the mouse (unpublished observation). To explore the selective pressures that might be acting on these non-coding RNAs, we downloaded human SNP data generated by The 1000 Genomes Project Consortium (2011) for the entire 15q11-q13 region (Figure 5A) and calculated Tajima's *D* statistic (Tajima, 1989) in a 10 Kb sliding window with a 2 Kb overlap. Negative values of Tajima's *D* indicate that there is an excess of low frequency polymorphisms (relative to the neutral expectation), whereas positive values indicate that there is an excess of intermediate-frequency polymorphisms.

The results show that the region bounded by *Snurf* and *Ube3a* has an excess of low frequency polymorphisms relative to the rest of the region (Figure 5B). To test whether this was the result of purifying selection, and therefore indicative of a conserved region, we downloaded two additional metrics (PhyloP and PhastCons) which measure, in distinct ways, the extent of nucleotide conservation across species for a genomic region. PhyloP measures conservation at individual sites without taking into account flanking regions. Positive values of this metric indicate conservation and negative values indicate faster divergence than expected under neutral drift. PhastCons, on the other hand, measures only conservation (probabilities between 0 and 1 with higher values indicating more conservation), and in addition takes into account flanking regions and hence is ideal for discovering functional elements. Notably, all genes located on human chromosome 15q11-q13, with the exception of *Atp10a*, are differentially expressed during human cortical neurogenesis.

In the region bounded by *Snurf* and *Ube3a*, PhyloP takes some of the largest negative values for the entire 15q11-q13 region, indicating that there is accelerated divergence of nucleotides across species within this region (Figure 5C). The values for PhastCons, however, do not deviate strongly from other regions, indicating that this region is not experiencing strong purifying selection across species (Figure 5D). Taken together, these results suggest that this region has undergone adaptive evolution in humans or experienced a selective sweep at some point in our recent past. To explicitly test whether the region bounded by *Snurf* and *Ube3a* exhibits a signature of adaptive evolution in humans, we applied a McDonald–Kreitman test (McDonald and Kreitman, 1991) on the numbers of polymorphic SNPs and divergent bases (relative to the chimpanzee) in the lincRNA sequences relative to silent-site polymorphism and divergence in the closely-linked *Snurf, Ube3a*, and *Atp10a* coding regions. After applying a Jukes-Cantor correction for sequence homoplasy to the divergent counts (Jukes and Cantor, 1969), we find strong evidence for an excess of divergence relative to polymorphism for the lincRNAs (*P* = 6.72 × 10^−8^; Table 1). The fraction of divergent bases that we estimate to be under positive selection in the lincRNAs, α (Smith and Eyre-Walker, 2002), is 0.764, an extremely high estimate. It remains possible that the excess of low frequency polymorphisms in the human data is the result of a selective sweep, and therefore SNP data from non-human species may help to shed light on the evolutionary forces acting on this region.

**Table 1:** **McDonald–Kreitman test for adaptive evolution of non-coding RNAs on human chromosome 15q11-q13**.

	**Polymorphic**	**Divergent**	**Total**
Neutral	56	24.07	80.07
Selected	102	186.25	288.25
Total	158	210.32	368.32

A 2 × 2 contingency table showing the numbers of polymorphic and divergent bases (relative to the chimpanzee) for three lincRNAs (Selected) bounded by Snurf and Ube3a, and for the silent sites in the coding regions of Snurf, Ube3a, and Atp10a (Neutral). The test shows that there is a significant excess of adaptive substitutions in the lincRNAs (P = 6.72 × 10^−8^, chi-squared test).

#### Selective conservation of lincRNA in large-brained species

**Figure 6:**
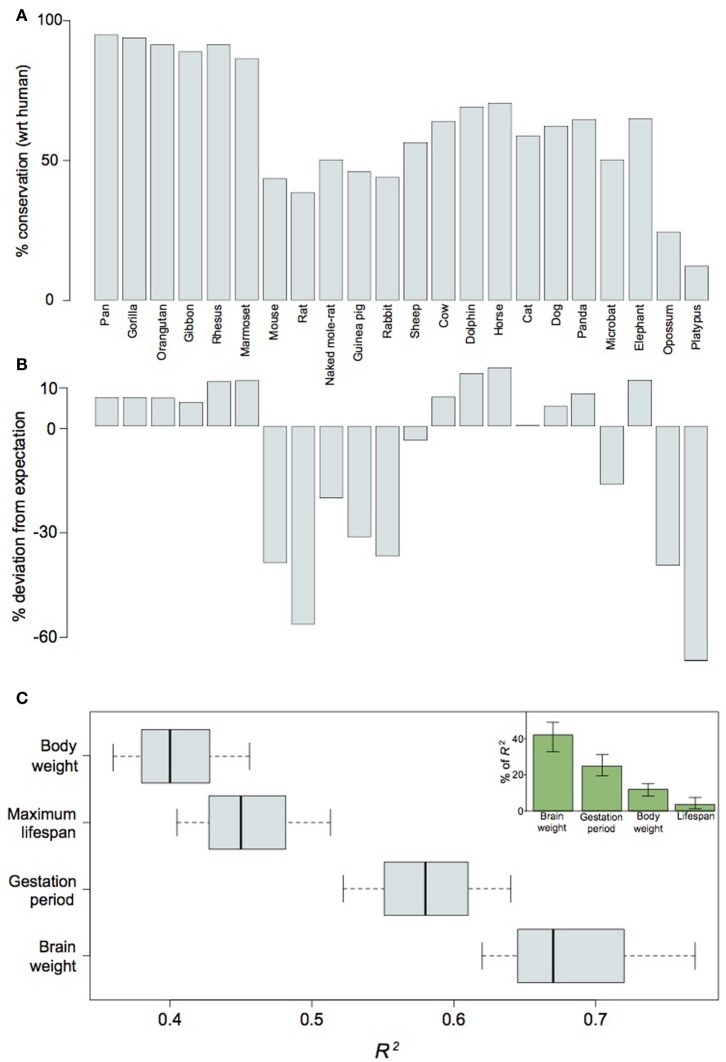
**LincRNA differentially expressed in human neocortical germinal zones are conserved with respect to their proximity to flanking genes between human and other large-brained mammalian species.** Barplots of absolute contextual conservation **(A)** and percent deviation of conservation from expectations-based on phylogenetic distance **(B)** for 161 lincRNA in 22 mammal species; Pearson's R^2^ scores for values in **(B)** as functions of brain weight, body weight, maximum lifespan, and gestation period **(C)**. The inset in **(C)** shows the relative contributions of each variable as a function of the total power of the combined variables (*R*^2^ = 82.5) for explaining values in **(B)**. Note that values in **(B)** may be better explained by brain weight than any other variable (*P* < 0.1, Welch's *t*-test).

Our analysis of the non-coding elements residing in the *Ube3a* imprinting domain reveals a potential importance for *cis*-regulated gene-expression in neocortical expansion. To assess whether lincRNA may be generally implicated in mammalian neocortical expansion, we took a broader perspective on differential gene regulation as it relates to neurodevelopment. Because lincRNA are often characterized as acting in *cis* (Ørom and Shiekhattar, 2011), the protein-coding neighborhood of the non-coding gene may be considered integral to its function. Therefore, we examined the genomic context of 161 lincRNAs expressed during the peak of human neurogenesis and observed protein-coding neighborhoods to be disproportionately conserved between humans and other large-brained mammalian species (Figures 6A,B). In fact, whether or not a species showed greater-than-expected conservation could be better predicted by brain weight than body weight, maximum lifespan, or even gestation period (Figure 6C). The conservation of such regions over tens of millions of years, as well as the relatively recent loss of the same regions in small-brained species, indicates an important role for lincRNA in the differential regulation, and especially timing, of neurodevelopment in large- vs. small-brained species. Heterochrony has been suggested as an expedient for brain evolution (Kelava et al., 2011) and lincRNAs may be one mechanism for achieving such changes in neurodevelopmental timing. We may point to the lincRNA located on chromosome 15q11-q13 as an example of a neurogenic regulator overrepresented in large-brained species and propose more generally that lengthening or shortening of the period of *in utero* maternal investment requires, at least where neurodevelopment is concerned, the conservation or loss, respectively, of *cis*-regulatory genomic regions.

## Parent-offspring conflict and parental-biased selection on neocortical expansion

According to Hamilton's Kin Selection Theory (Hamilton, 1964a,b), the transfer of nutrients from mother to fetus may be viewed as evidence of altruism, because increased maternal investment reduces the fitness of the mother while increasing the fitness of the fetus. But this is not strictly the case. Early in development, fetal trophoblast cells invade the maternal endometrium and remodel the maternal vasculature (Angiolini et al., 2006), effectively wresting control of maternal-to-fetal blood flow from the mother. In polyandrous societies, which dominate in mammals, the fetus may be seen as a proxy for the father, his lone representative, due to the fact that the paternal genome will only pass through the patriline. So in order to optimize the fitness of patrilineal progeny, paternally inherited alleles favor increased maternal investment (i.e., promote growth) compared to maternally inherited alleles; and the allocation of maternal resources to the fetus, therefore, is less an altruistic arrangement between mother and fetus—except perhaps where hemostatic regulation is concerned—but an intragenomic conflict between paternal and maternal interests (Moore and Haig, 1991; Haig, 2010). The incremental evolution of imprinted domains and increased risk of neurodevelopmental disease associated with parent-of-origin-linked gene expression suggest, firstly, that this conflict is not brokered exclusively in growth hormones at the fetal-placental interface and, secondly, that both parents evolve selfish strategies that lead to higher risk of disease for the population at large, reminiscent of Hardin's “tragedy of the commons” (Hardin, 1968), in the evolutionary arms race to regulate fetal development.

### Incremental evolution of imprinted domains and its neurodevelopmental effects

Imprinted regions manifest as the epigenetic silencing of one allele and high expression of the other. It is unlikely that the levels of expression observed in imprinted regions evolved suddenly, but that the large detectable differences in parental allelic expression are the consequence of a gradual gene-expression arms race (Mills and Moore, 2006; Moore, 2012). Indeed, many genes show expression bias for one allele, but not complete silencing of the other allele (Gregg, 2010). Following many generations of selection for increased expression at each parental site, the difference between the alleles may be nearly unchanged, but the absolute level of expression at each allele will have increased dramatically, thereby ministering a more striking maternal- or paternal-biased phenotype when the opposite allele is imprinted. Because evolutionary models of brain-specific imprinted genomic regions have been limited (O'Connell et al., 2010; McCole et al., 2011), it is difficult to surmise whether a maternal- or paternal-biased phenotype has been under selection or even what that phenotype may be. While experimental evidence for the phenotypic effects of imprinted domains has been conducted in the mouse, our hypothesis predicts these effects will be contrapositive to those present in the human and other large-brained species. A three-fold increase in human compared to mouse imprinted genes expressed during cortical neurogenesis supports this claim (see Figure 1). Therefore, any phenotypic evidence presented for the disruption or manipulation of imprinted domains in the mouse cannot, we contend, be extrapolated to the human. As a first step in investigating a mammalian-wide evolutionary picture of parent-of-origin-linked genes, we present evidence that imprinted genomic regions have been under pervasive purifying selection, indicative of both the role of these genes in disease (Blekhman et al., 2008; Cai et al., 2009) and the importance of non-coding neighbors in their temporal-, tissue-, and species-specific regulation of expression. The phenotypic effects of an evolutionary arms race between maternal and paternal alleles in humans is further evident in the numerous pathologies that present themselves when parent-of-origin-linked gene expression is imbalanced during neurodevelopment. Finally, the large metabolic costs of neurodevelopment during gestation advance the brain as a likely target for intragenomic conflict over resource allocation, even if pathways of maternal-fetal metabolic regulation are not directly the subjects of selection.

### Neocortical expansion: tragedy of the commons

Monoallelic gene expression increases the risk of disease, because the functional haploidy associated with genomic imprinting increases the chances of an individual experiencing the phenotypic effects of a recessive deleterious mutation. While these mutations may have little-to-no effect when heterozygous at an unimprinted locus, they present severe fitness effects at an imprinted locus, where the functional haploidy associated with imprinting gives a recessive deleterious mutation a 50% chance of presenting itself. This introduces a scenario in which both parents have evolved a system in which, if either parent's gene is not sufficiently expressed and its phenotype successfully passed through its respective lineage, then the fetus will suffer (often lethal) developmental defects. This is an evolutionary Tragedy of the Commons because ever-more selfish strategies will inevitably evolve at the expense of the fitness of the whole population.

So why would genomic imprinting have evolved an important role in mammalian brain development and why should we expect selection in genomically imprinted domains? The evidence presented here for pervasive purifying selection on imprinted neurogenic genes suggests that parent-offspring conflict has affected these genes by acting selectively on non-coding regulators of their expression, thus influencing their expression without affecting their overall function. Indeed, our analysis of the non-coding domain on chromosome 15q11-q13 and, more broadly, our observation that putatively *cis*-acting lincRNAs are selectively conserved in large-brained mammalian species predict a requisite role for non-coding regions in evolving and developing a large brain. Of course, the general positive correlation of lincRNA conservation with not only brain weight, but also body weight, lifespan, and gestation period (see Figure 6C) may suggest that lincRNAs are selectively lost along lineages leading to smaller species with shorter life-histories, evincing a broad mechanistic role for lincRNAs in facilitating evolutionary changes to developmental timing and, more generally, underscoring the constraining role of the rate of meiotic recombination in the evolutionary history of a species (Romiguier et al., 2013). But even though a higher rate of meiosis may increase the frequency of meiotic errors and therefore the likelihood of losing lincRNAs in small species, the small effective population size of most large species would also hasten the loss of weakly selected genomic regions. As such, the conservation of lincRNAs along any lineage should be evidence of strong selection and function.

Because workers have shown that positively selected genes affecting human-specific phenotypes are more likely to be expressed in fetal compared to adult tissue (Uddin et al., 2008), it follows that genomically imprinted domains should show signatures of selection along mammalian lineages with ostensible evidence for maternal- or paternal-biased phenotypes. We posit that the mammalian, and especially human, neocortex presents such a phenotype; and that further computational and experimental studies on imprinted domains will help us understand how parent-offspring conflict has influenced maternal- or paternal-biased brain phenotypes at the genomic level.

### Conflict of interest statement

The authors declare that the research was conducted in the absence of any commercial or financial relationships that could be construed as a potential conflict of interest.

## Appendix

**Table A1:** **Selection statistics for imprinted neurogenic genes in human and mouse**.

**Gene**	**Positive selection (*p* < 0.1)**	**Negative selection (*p* < 0.1)**	**Pervasive diversifying selection (posterior probability > 0.9)**	**Pervasive purifying selection (posterior probability > 0.9)**	**Episodic diversifying selection**	**Mean dN/dS**	**Human neurogenic expression**^**a**^	**Mouse neurogenic expression**^**a**^	**Allelic expression**
C10orf93	49	1022	4	1576	173	0.317	Yes	No	Maternal
C16orf57	3	138	1	172	3	0.211	Yes	No	Maternal
CDK4	0	101	0	146	5	0.171	Yes	No	Maternal
COPG2	0	194	0	279	5	0.082	Yes	No	Paternal
DIRAS3	5	58	1	118	18	0.374	Yes	No	Maternal
E2F7	1	175	0	226	10	0.366	Yes	No	Maternal
FERMT2	0	477	0	626	6	0.027	Yes	No	Paternal
GABRA5	4	313	2	432	16	0.108	Yes	No	Paternal
GATM	0	202	1	320	10	0.109	Yes	No	Maternal
GLI3	8	896	6	1193	39	0.151	Yes	No	Maternal
HES1	0	130	0	217	6	0.091	Yes	No	Paternal
HSPA6	0	201	0	385	18	0.232	Yes	No	Maternal
HTR2A	1	279	0	367	16	0.131	Yes	No	Maternal
IMPACT	4	127	3	191	13	0.207	Yes	No	Biallelic
L3MBTL4	7	227	2	335	42	0.316	Yes	No	Paternal
LDB1	0	158	0	298	6	0.064	Yes	No	Maternal
MEST	0	194	0	279	5	0.082	Yes	No	Paternal
NKAIN3	0	105	0	149	3	0.098	Yes	No	Paternal
NTM	0	189	1	294	4	0.053	Yes	No	Paternal
OTX1	0	187	0	311	4	0.058	Yes	No	Maternal
PEG3	9	141	7	162	54	0.574	Yes	No	Paternal
PEX10	1	166	0	256	3	0.211	Yes	No	Maternal
PRDM16	10	452	4	796	33	0.161	Yes	No	Paternal
PRIM2	3	267	2	354	21	0.238	Yes	No	Paternal
PTPN14	1	739	1	1053	15	0.088	Yes	No	Maternal
RB1	7	418	3	702	23	0.152	Yes	No	Maternal
SALL1	8	668	5	950	20	0.132	Yes	No	Maternal
SDHD	1	68	0	83	9	0.306	Yes	No	Paternal
SGCE	0	251	0	349	9	0.051	Yes	No	Paternal
SNURF	0	105	0	152	1	0.186	Yes	No	Paternal
SOX8	2	190	0	363	4	0.116	Yes	No	Paternal
UBE3A	0	510	1	778	7	0.069	Yes	No	Maternal
USF1	0	154	0	258	4	0.071	Yes	No	Maternal
ZFP36L2	6	409	1	744	39	0.263	Yes	No	Maternal
AXL	6	415	2	692	23	0.157	Yes	Yes	Maternal
CDKN1C	1	36	3	70	16	0.453	Yes	Yes	Maternal
DLX5	0	117	0	210	4	0.096	Yes	Yes	Maternal
PON2	4	154	5	247	21	0.229	Yes	Yes	Unknown
ERBB3	4	781	2	1163	34	0.131	No	No	NA
FMR1	12	219	32	316	47	0.198	No	No	Maternal
SYCP3	6	125	9	208	50	0.348	No	No	Unknown
Atp10a	22	673	6	1070	38	0.214	No	Yes	Maternal
Gabrb3	0	263	0	346	5	0.039	No	Yes	Unknown
Grb10	1	384	1	503	15	0.138	No	Yes	Maternal
Nnat	0	27	0	65	1	0.04	No	Yes	Paternal
Osbpl5	10	361	2	630	19	0.181	No	Yes	Maternal
Peg10	4	156	3	239	26	0.162	No	Yes	Paternal
Peg12	3	55	1	117	11	0.349	No	Yes	Paternal
Plagl1	2	186	2	288	19	0.277	No	Yes	Paternal
Snrpn	0	100	1	148	1	0.18	No	Yes	Paternal
Usp29	17	96	3	105	91	0.722	No	Yes	Paternal

a Differentially expressed in a neocortical germinal zone.
